# The scope of interventional radiology training for South African radiology registrars

**DOI:** 10.4102/jcmsa.v4i1.374

**Published:** 2026-08-17

**Authors:** Zainul-abidin Shaik, Yusuf Parak, Michelle Da Silva, Richard D. Pitcher

**Affiliations:** 1 Department of Medical Imaging and Clinical Oncology, Faculty of Medicine and Health Sciences, Stellenbosch University, Cape Town, South Africa

**Keywords:** radiology, registrar training, interventional radiology, radiology education, radiology curriculum

## Abstract

**Background:**

Interventional radiology (IR) is an important component of radiology practice in South Africa (SA). However, there is no standard national IR curriculum. Little is known about IR teaching across SA’s nine medical schools, of which six offer a 4-year and three offer a 5-year postgraduate radiology training programme. The study assessed procedural performance scope among SA postgraduate radiology trainees across key IR procedures.

**Methods:**

This retrospective, cross-sectional study evaluated candidates who completed SA postgraduate radiology training and sat the final FC Rad Diag(SA) examination (2020–2024). Respondents reported practical performance across 14 image-guided procedures (ultrasound [US] = 4; US + fluoroscopy [US + F] = 5; computed tomography (CT) = 3; fluoroscopy with digital subtraction angiography [F + DSA] = 2). Descriptive statistics were generated overall, by modality and by training period.

**Results:**

A total of 38 candidates responded. Four (11%) performed all, and one (3%) just two procedures. Experience varied widely (average eight procedures); 47% (n = 18) performed ≤ 7. Overall performance rates by modality were: US-guided 91%, US + F 49%, CT-guided 41%, and F + DSA 41%. The 4-year (*n* = 17; mean = 7) and 5-year programmes (*n* = 21; mean = 9) afforded similar rates for US-guided procedures (90% vs. 92%). In contrast, 5-year programmes showed higher rates for US + F (62% vs. 34%), CT-guided (57% vs. 22%), and F + DSA (45% vs. 35%).

**Conclusion:**

The marked disparity in exposure to IR procedures among SA general radiology trainees underscores the need for standardised IR training.

**Contribution:**

To the best of our knowledge, this is the first study to document the procedural scope of IR training in SA. As such, it provides unique insights that can inform curriculum development and workplace-based assessments across the radiology teaching platform.

## Introduction

Interventional radiology (IR) is a dynamic, rapidly evolving field of diagnostic radiology. It utilises advanced imaging to guide minimally invasive procedures, providing patients with effective, less invasive treatment options.^1^

The origins of IR are rooted in early advancements in radiological contrast agents and vascular imaging, culminating in Seldinger’s groundbreaking percutaneous catheter technique in 1953.^2^ The field transitioned from purely diagnostic to therapeutic in 1964 when Charles Dotter performed the first intentional percutaneous angioplasty.^3^ Over the subsequent decades, rapid technological developments, including high-resolution ultrasound, computed tomography (CT) guidance, and advanced therapeutic devices, expanded the scope of IR into a mainstream medical discipline.^4,5,6,7,8,9^ Today, the utilisation of minimally invasive, image-guided interventions has expanded rapidly worldwide, significantly reducing patient hospitalisation times, procedural risks, and recovery periods.^10,11,12,13^

However, the integration of IR into general radiology curricula faces significant challenges, including a lack of standardised training pathways, as well as high equipment and disposable costs.^14,15^ Furthermore, interdisciplinary competition for IR procedures contributes to limited exposure for radiology registrars, impacting training.^16,17^ These obstacles result in considerable variation in the procedures that trainees encounter across institutions.^15^

Internationally, general radiology curricula integrate IR to different extents. Several systems define minimum procedural expectations for all general radiologists. In the United Kingdom, the Royal College of Radiologists integrates IR into general registrar training and specifies core competencies, notably ultrasound-guided fine-needle aspiration (FNA) and core biopsy, vascular access, drainages, and fluoroscopically guided nephrostomy.^18,19^ In Australia and New Zealand, the Royal Australian and New Zealand College of Radiologists requires graded exposure that culminates in core image-guided skills, including ultrasound-guided biopsies and drainages and safe line or port access, with further fluoroscopic skills encouraged.^20,21^ In Canada, protected interventional and vascular rotations within diagnostic radiology emphasise competence in ultrasound-guided tissue sampling, drainages, and basic catheter skills, with fluoroscopic procedures acquired according to programme capacity.^22,23^ In the United States, diagnostic radiology programmes ensure residents can perform common image-guided procedures such as biopsies, drainages, and access.^24,25,26^

In South Africa (SA), there are nine accredited postgraduate training centres for general radiology; six offer a 4-year and the remainder a 5-year curriculum. Interventional radiology training is therefore not standardised across programmes. At Chris Hani Baragwanath Academic Hospital, one of the main teaching hospitals of the University of the Witwatersrand, the top three IR procedures in 2021 were percutaneous transhepatic biliary drainage (PTBD) (26%), bronchial artery embolisation (10%), and pigtail catheter insertion (8%).^27^ At Tygerberg Hospital, the primary teaching hospital of Stellenbosch University, the most common procedures over the 2017–2019 period were aorto-bifemoral diagnostic angiography (25%), aorto-bifemoral interventional angiography (12%), and unilateral nephrostomy (11%).^28^ These findings may not fully represent procedures performed specifically by radiology registrars, as they focus on fluoroscopic-guided procedures and are confined to two hospitals, with potential contributions from other specialties.

A clearer understanding of the current state of IR training in SA is important. It would identify variations in registrar exposure and experience across training institutions. Such information may assist stakeholders in informing future training policy and in considering approaches to curriculum alignment and standardisation, particularly in resource-constrained settings.

This study aims to assess IR training within general radiology training programmes in SA.

## Research methods and design

### Study design

This study employed a retrospective, questionnaire-based, cross-sectional design.

### Setting

The study was facilitated by the Colleges of Medicine of South Africa (CMSA), the nationally accredited examining body responsible for the unitary exit examinations for all medical specialties in SA.

### Study population

The study was limited to those who had completed formal postgraduate radiology training at a South African institution and who sat the final fellowship examination of the College of Radiologists of the CMSA (FC Rad Diag[SA] Part II) from 2020 through 2024, as recorded in the official CMSA database. FC Rad Diag(SA) Part II candidates outside the specified period were excluded.

### Data collection

The Registrar of the CMSA searched the official CMSA database to identify all candidates who wrote the FC Rad Diag(SA) Part II from 2020 to 2024. Utilising contact details provided at the time of exam registration, the CMSA emailed prospective participants, including the survey link and details of the purpose, format and voluntary nature. Contact details of the principal investigator were provided for potential queries. Participants were given 1 month to complete the survey, with weekly reminders.

The questionnaire (Online Appendix 1) evaluated exposure across 14 specific IR procedures. The procedure list was curated and validated for relevance by the Head of IR at a South African tertiary training hospital. The selection was designed to capture a representative spectrum of non-vascular and vascular image-guided procedures routinely performed within the South African public healthcare sector. For each procedure, the extent of training was defined as the participant’s maximum achieved level of practical involvement, quantified using a 5-point ordinal scale: ‘I have had no exposure’, ‘I have observed’, ‘I have assisted a senior’, ‘I have performed with assistance’, and ‘I have performed without assistance’.

Procedures were stratified by imaging modality as those requiring (1) ultrasound alone (*n* = 4), (2) ultrasound with fluoroscopy (US + F) (*n* = 5), (3) CT (*n* = 3), and (4) fluoroscopy and digital subtraction angiography (F + DSA) (*n* = 2).

### Data analysis

Responses were captured on a customised Microsoft Excel (Microsoft, Redmond, WA, United States) spreadsheet. The de-identified data were transferred to Stata Version 18 (StataCorp, College Station, TX, United States) for further analysis. Categorical variables were summarised as percentages. To ensure a clinically meaningful assessment of practical capability, the five original survey response categories were aggregated into a binary variable for final analysis. Responses indicating ‘I have performed with assistance’ or ‘I have performed without assistance’ were stratified as ‘performed’. This threshold was chosen to represent active, hands-on procedural experience. Conversely, responses of ‘I have had no exposure’, ‘I have observed’, or ‘I have assisted a senior’ were stratified as ‘not performed’, as they reflect passive or strictly supportive exposure rather than primary execution of the skill. To characterise variations between the training cohorts, absolute differences in procedural performance rates between the 4-year and 5-year programmes were evaluated descriptively. This approach aligns with principles in the medical education literature, where quantitative gaps in procedural exposure are utilised to identify training variances and guide curriculum development, even in the absence of statistical power calculations typical of small cohort studies.^29,30^

### Ethical considerations

Approval was obtained from the Health Research Ethics Committee of Stellenbosch University (Reference No: S24/08/212) and the Education Research Related Working Group of the Education Committee of the CMSA. Data were collected anonymously, with no information regarding individual identity or training institutions.

## Results

### Overview

The CMSA recorded 189 candidates who sat the FC Rad Diag(SA) Part II examination between 2020 and 2024. The survey yielded responses from 47 individuals, representing an overall response rate of 25%. Of these, nine respondents had not completed general radiology training and were excluded. The remaining 38 respondents (20% of the total candidate cohort) had completed general radiology training and were included in the analysis. Of these, 21 (55%) had followed the 5-year and 17 (45%) the 4-year programme.

### Scope of procedural exposure (Table 1)

Thirty-eight (*n* = 38) candidates responded. Four (*n* = 4; 11%) had performed all, and one (*n* = 1, 3%) just two procedures. This absolute volume of experience varied widely, with an overall average of eight procedures, and nearly half (*n* = 18/38; 47%) performing seven or fewer. Overall procedural performance rates across the modalities were 91% for ultrasound-guided, 49% for ultrasound with fluoroscopy, 41% for CT-guided, and 41% for digital subtraction angiography. The 4-year (*n* = 17; mean = 7 procedures) and 5-year programmes (*n* = 21; mean = 9 procedures) afforded similar overall performance rates for ultrasound-guided procedures (90% vs. 92%). In contrast, the 5-year programmes demonstrated higher performance rates for ultrasound with fluoroscopy (62% vs. 34%), CT-guided procedures (57% vs. 22%), and digital subtraction angiography (45% vs. 35%).

**TABLE 1:** Number and proportion of procedures ‘performed’ overall and by training duration.

Procedures	Overall		4-year programme (*n* = 17)		5-year programme (*n* = 21)	
	*n*	%	*n*	%	*n*	%
**US guided**						
FNA	38	100	17	100	21	100
Biopsy	35	92	16	94	19	90
Drainage	33	87	15	88	18	86
Pigtail insertion	32	84	13	76	19	90
Average	-	91	-	90	-	92
**USF guided**						
Nephrostomy	25	66	9	53	16	76
PTC and drainage	22	58	8	47	14	67
Cholecystostomy	19	50	6	35	13	62
PTC and stent	14	37	3	18	11	52
JJ stent insertion	14	37	3	18	11	52
Average	-	49	-	34	-	62
**CT guided**						
Biopsy	16	42	4	24	12	57
Drainage	16	42	4	24	12	57
FNA	15	39	3	18	12	57
Average	-	41	-	22	-	57
**DSA**						
Angiography: diagnostic	16	42	6	35	10	48
Angiography: coil and embolisation	15	39	6	35	9	43
Average	-	41	-	35	-	45

US, ultrasound; USF, ultrasound and fluoroscopy; FNA, fine-needle aspiration; CT, computed tomography; DSA, digital subtraction angiography; PTC, percutaneous transhepatic cholangiogram.

## Discussion

Certain interventional procedures are widely recognised in the literature as basic for general radiologists. These include image-guided FNA, core biopsies, and percutaneous drainage. Furthermore, fluoroscopy-guided procedures including diagnostic angiography, cholangiography, and nephrostomy are regarded as essential in emergency and resource-limited settings because of their clinical impact, frequent use and lack of established IR sub-specialty services. These procedures should arguably be regarded as foundational competencies for general radiologists in SA.^19,22,31^

Our finding that only 11% of respondents had performed all stipulated interventional procedures, and that registrars on average had experience with approximately half of the procedures assessed, highlights substantial variability in procedural exposure among this cohort and suggests a need for further investigation into curriculum standardisation. Furthermore, while these data are indicative rather than definitive, the observation that some respondents had performed as few as 2 of the 14 procedures highlights a potential vulnerability regarding underexposure to essential procedures prior to independent practice.

Several studies have shown that longer training duration is associated with improved clinical competence and educational outcomes.^32,33,34^ Comparisons between the 4-year and 5-year training programmes reveal that both curricula achieve near-universal exposure for the core ultrasound-guided interventions. For example, ultrasound-guided FNA was performed by all registrars in both groups, and biopsy and centesis rates exceeded 80% in each. In contrast, essential fluoroscopy-guided procedures showed consistently higher exposure in the 5-year group, with marked descriptive differences in absolute rates, such as diagnostic angiography (19%), percutaneous transhepatic cholangiogram (PTC) with drainage (19%), nephrostomy (23%), cholecystostomy (26%), PTC and stent (34%), JJ stent insertion (34%) and all CT guided procedures (> 34%). Differences of this magnitude are educationally meaningful for skills that are critical to emergency and on-call practice, and they point to the practical advantage of additional training time in securing competency.^29,30^ This pattern supports the view that time available for rotation and repetition, rather than case availability, is a limiting factor for registrar proficiency in essential procedures.

Currently, there is no standardised IR curriculum for general radiology programmes in SA. In contrast, many high-income countries have implemented structured guidelines to assess interventional competency among general radiology registrars. Studies have shown that standardised curricula improve educational outcomes, trainee preparedness, and quality of patient care – both globally and within SA.^35,36,37^ These frameworks allow for objective, unified evaluation across institutions, rather than subjective assessments based on institution-specific procedural volume.

In the United States, general radiology registrars are only required to perform 25 image-guided biopsies and drainages to meet training requirements.^25^ In comparison, South African registrars often perform not only basic procedures but also advanced interventions. The key distinction is that in high-income countries, subspecialised IR services are well established, reducing the necessity for comprehensive IR competency among general diagnostic radiologists. In contrast, within the South African context, formal subspecialty IR infrastructure and dedicated training fellowships have only recently been established. This baseline limitation results in a critical shortage of dedicated subspecialist skills nationally. Consequently, in emergency, rural, and resource-constrained local settings, general radiologists are frequently called upon to bridge this gap by performing a broader spectrum of essential non-vascular and vascular interventions to ensure timely patient care.^38^ Given these system constraints, local academic institutions must decide whether to invest in strengthening IR competency among general radiology registrars or shift resources towards developing dedicated subspecialty IR services – aligning with international trends.

There are limited published data on IR training infrastructure across accredited radiology training institutions in SA. Future research could assess institutional IR capacity, including physical infrastructure and the availability of trained IR staff. In addition, it is important to clarify which clinical specialties are performing these procedures, as some are also undertaken by other disciplines such as urology and cardiology. Such research may further serve as a proxy marker for inequities in radiology registrar training more generally, extending beyond IR alone.

This study surveyed a nationwide cohort of individuals who had completed radiology registrar training, offering a broad overview of training experiences across diverse institutions. Interpretation must be approached cautiously as local data on IR procedural exposure are limited to two institutions.^27,28^ The relatively low response rate (20%, *n* = 38) introduces potential non-response bias. However, the marked internal variance observed across procedural modalities, ranging from near-universal performance in basic ultrasound interventions to profound disparities in complex fluoroscopic and CT-guided procedures, suggests that the data capture genuine underlying structural variations in training rather than systemic non-response bias alone. Reliance on self-reported data also introduces potential recall bias. However, because primary procedural execution represents a discrete, practical milestone within clinical residency, the probability of subjective misclassification regarding independent performance remains low. In addition, institutional variations in training structures, such as differences in equipment, regional case-mix, and local supervision dynamics, were not fully accounted for. Failing to adjust for these baseline institutional differences means that training duration is likely not the sole factor driving the observed variances. Consequently, these results should be interpreted as indicative of training trends rather than a definitive causal assessment, highlighting that further work is required to establish the precise impact of institutional infrastructure on programme-specific exposure.

## Conclusion

This national survey highlights that while South African radiology registrars achieve strong competency in ultrasound-guided procedures, exposure to key fluoroscopic and CT-guided interventions remains variable, with meaningful advantages observed in the 5-year programme. Addressing these gaps through structured curricula, protected teaching time, and targeted rotations could help ensure that all graduating radiologists are equipped with essential interventional skills for independent practice.
